# Metabolic and Nutritional Aspects in Paediatric Patients with Klinefelter Syndrome: A Narrative Review

**DOI:** 10.3390/nu14102107

**Published:** 2022-05-18

**Authors:** Chiara Mameli, Giulia Fiore, Arianna Sangiorgio, Marta Agostinelli, Giulia Zichichi, Gianvincenzo Zuccotti, Elvira Verduci

**Affiliations:** 1 Department of Pediatrics, Vittore Buzzi Children’s Hospital, University of Milan, 20154 Milan, Italy; giulia.fiore@unimi.it (G.F.); arianna.sangiorgio@unimi.it (A.S.); marta.agostinelli@unimi.it (M.A.); giulia.zichichi@asst-fbf-sacco.it (G.Z.); gianvincenzo.zuccotti@unimi.it (G.Z.); elvira.verduci@unimi.it (E.V.); 2 Department of Health Sciences, University of Milan, 20142 Milan, Italy

**Keywords:** Klinefelter syndrome, children, bone metabolism, hypogonadism, body composition, diabetes, metabolic syndrome, cardiovascular risk, hormonal replacement therapy

## Abstract

Klinefelter syndrome is the most common sex chromosomal aneuploidy in males. It is well known that patients with this syndrome have greater mortality and morbidity compared to the general population due to cardiovascular diseases and endocrine metabolism disorders. This augmented risk is due both to hypogonadism and to the syndrome itself. Therefore, correct hormonal replacement therapy and early primary prevention are crucial to these patients. Even though different studies are available on this topic in adult patients, only a few authors have focused on the paediatric population. Thus, in this narrative review, we report the current knowledge of metabolic and nutritional aspects in children with Klinefelter syndrome.

## 1. Klinefelter Syndrome: Clinical Characteristics and Diagnosis

Klinefelter syndrome (KS) is the most common sex chromosomal aneuploidy in males. It is characterized by an additional X chromosome, which leads, in most cases, to a karyotype of 47 XXY [1,2].

The prevalence of KS is estimated between 1 in 448 to 1 in 917 male births [1,3,4]. The prevalence seems to have increased during the last few decades. This could be partially explained by increasing maternal age, poorer sperm quality, environmental changes and decreased elective termination for prenatally diagnosed cases [1,4].

The supernumerary X-chromosome originates in 50% of the cases from the mother and in the other 50% of the cases from the father [1,4]. In both situations, it is due to a non-disjunction event during parental gametogenesis or post-zygotic non-disjunction during early embryonic mitotic divisions. In 90% of the patients with KS, the karyotype is a typical 47 XXY, while 7% of the patients have mosaicism. The other 3% have rare variants, such as 48 XXXY or 48 XXYY. These variants with two extra sexual chromosomes are extremely rare (1:18,000–1:100,000) and are usually characterized by a more severe phenotype [1,5,6]. The presence of an extra chromosome is extremely important to determine the phenotype. In fact, some genes are shared between the X and Y chromosomes. These mutual genes regulate other genes that are overexpressed in KS and lead to the phenotype [4]. Additionally, there is an important phenotype-modifying marker to be considered: the (CAG)n repeat length in the androgen receptor gene. A higher number of repetitions is associated with height, gynecomastia and small testes [4].

Historically, KS was first described in 1942 by Dr Harry Klinefelter, who described a case series of men characterized by small testes, tall stature, narrow shoulders, broad hips, sparse body hair, gynecomastia and azoospermia [1,4,7]. Several studies have been performed since the 1940s and the knowledge regarding KS has largely increased. Mild-to-moderate cognitive deficits have been added as symptoms of KS [8]. Moreover, we currently know that an alternative phenotype with fewer clinical features is widely represented [7].

Hypogonadism is a central element of KS. The testicular underdevelopment and dysfunction could be as early as infancy [2]. Already during mini-puberty in some patients, there is a blunted testosterone surge compared to their peers [4]. Germ cells are fewer than normal and their deficit worsens while the patient grows until in adulthood, when spermatogenesis is almost suppressed [7]. The hypothalamic–pituitary–gonadal axis (HPG axis) has normal activity; therefore, it is quiescent during childhood and is activated at the beginning of puberty. In many cases, the pubertal onset is normal [4,7]. Nevertheless, the testes usually reach a maximal volume of 10 mL and then decrease to 4 mL [7].

The signs and symptoms of KS vary depending on age. In a few cases, newborns can show signs of hypogonadism, such as a micropenis, cryptorchidism and hypospadias [9]. In the case of a micropenis, treatment with testosterone should be suggested [7]. Symptoms that lead to a diagnosis during childhood are usually underdevelopment of genitalia, hypotonia, developmental delays and learning and behaviour problems. In particular, 75% of children with KS present speech–language delay, 50% have motor-skills delays and between 36 and 63% are diagnosed with Attention Deficit Hyperactivity Disorder (ADHD) [1]. Too often, when the presenting symptoms are neurological disabilities, there is a delay in the diagnosis of KS.

Patients with KS are diagnosed during adolescence due to small testicles, gynecomastia or delayed puberty. However, often, the diagnosis is made during adulthood during investigations for infertility or in patients who present signs and symptoms of hypogonadism [1].

To date, we know there is an important discrepancy between the known prevalence of KS and the rate of clinical diagnosis [4]. In fact, only 25–35% of the patients with KS are diagnosed during their life, while most of the patients are left without a diagnosis [1,7]. Considering the males who received a diagnosis, approximately 10% of the cases are diagnosed during prenatal screening, 6% in childhood or adolescence and 19% in adulthood [1].

During adolescence, hormonal exams could be useful for diagnosis. In fact, testosterone may rise normally at the beginning of puberty; however, it usually decreases to lower-normal or subnormal levels. In response to this phenomenon, luteinizing hormone (LH) and follicle-stimulating hormone (FSH) rise 1–2 years after the beginning of puberty. Moreover, inhibin B (INHB) and anti-Mullerian hormone (AMH) are low or undetectable [1]. Once KS is suspected based on clinical and hormonal data, a genetic test is mandatory in order to make a diagnosis [1].

If KS is found early in infancy or even at the prenatal tests, the patient should undergo an adequate follow-up [4]. In particular, it is extremely important that children with KS are tested with periodical neuropsychological evaluations. Moreover, periodical endocrinological visits with pubertal examinations and hormonal dosages are suggested, starting from 10 years old or at the first sign of puberty, depending on which comes first.

Currently, there are no universal guidelines that indicate when androgen replacement should be begun. Some authors suggest waiting until signs or symptoms of hypogonadism appear. Others prefer to start low testosterone doses at the first abnormal rise of LH. Testosterone is administered with depot injection or topical gel. In order to preserve the possibility of paternity, it is crucial to refer the patient to a centre specialized in advanced reproductive technology, in particular in testicular sperm extraction [1].

It is extremely important to remind that KS is not only about hypogonadism and neurodevelopment.

Different studies have focused on the long-term consequences of KS, showing that Klinefelter patients have greater morbidity and mortality [10,11]. The median survival rate is 2 to 5 years lower compared to peers. The augmented mortality rate is connected especially to cardiovascular diseases and endocrine metabolism disorders [3,12]. In fact, KS could have effects on body composition, insulin sensitivity, bone metabolism and cardiovascular morbidity, with an increased risk of diabetes, metabolic syndrome, osteoporosis and cardiac disease [7,13].

For these reasons, in this narrative review, we focus on the metabolic and nutritional aspects of KS, particularly in the paediatric population. These aspects are extremely important considering that early diagnosis will be more frequent due to prenatal tests. Therefore, a correct approach to nutrition and hormonal therapy in children and adolescents with KS will be necessary in order to reduce the cardiometabolic risk and, consequently, morbidity and mortality.

## 2. Methods

A narrative review was performed starting on 29 January 2022 [14]. The most relevant original scientific papers, clinical trials, meta-analyses and reviews published in the English language, on a specific topic, were reviewed. The following keywords (alone or in combination) were considered: “Klinefelter syndrome”, “children”, “bone metabolism”, “hypogonadism”, “body composition”, “diabetes”, “metabolic syndrome”, “cardiovascular risk”, “hormonal replacement therapy”. Approximately 87 papers were considered. The electronic databases PubMed, Scopus, EMBASE and Web of Science were used for this research. The resulting draft was discussed with all co-authors. The final version was then recirculated and approved by all.

## 3. Metabolic Aspects

It is well known that adult patients with hypogonadism present several metabolic alterations independently from KS. In fact, in the general adult male population, hypogonadism is associated with abnormal body composition, metabolic syndrome, insulin resistance, diabetes, elevated cardiovascular risk and osteoporosis [7]. Moreover, there is a vicious circle between abdominal adiposity and insulin resistance, with abdominal adiposity causing insulin impairment [7,15]. In addition, insulin resistance usually worsens hypogonadism by lowering hypothalamic activity and impairing Leydig cell function [12].

Despite the clear connection between hypogonadism and metabolic alterations, in patients affected by KS, this interaction is extremely complex because hypogonadism is not the only factor that should be considered [4]. In fact, metabolic abnormalities partially subsist due to the genetic disease itself [12].

Few studies have evaluated the metabolic aspects in paediatric patients affected by Klinefelter syndrome so far. Table 1 summarizes the state of the art in the paediatric population to date.

### 3.1. Adiposity

There is a growing clinical and scientific interest in the metabolic alterations and the unfavourable changes in body composition present in KS patients since childhood. They typically have a tall stature, perhaps caused by SHOX duplication, and specific anthropometric findings, such as increased length, width and hip circumference [26].

As mentioned beforehand, androgens play an important role in regulating adipogenesis and body fat distribution. Hypogonadism, common in adults with KS, has been found to be an independent risk factor for increasing truncal adiposity and diabetes in men with normal karyotypes [27,28,29].

Nevertheless, some authors reported an increased deposit of body fat, by the age of ten years in patients with KS, measuring subscapular and triceps skinfolds [30]. In prepubertal boys with KS, age 4 to 12 years, an increase in waist circumference and an increased risk of metabolic syndrome have been demonstrated [15,17].

Furthermore, in a recent cross-sectional study, Aksglaede et al. showed that an unbalanced body composition among KS adults may already be present before puberty. In prepubertal subjects, with a median age of 11 years old, an increasing body fat mass (BFM), height and BF% were found upon a DEXA scan despite age-normal BMI, lean body mass (LBM) and weight for age, compared with normal boys, probably due to an unfavourable muscle-to-fat ratio. Moreover, increased BFM was demonstrated in both testosterone-treated and untreated KS children [20]. However, Davis et al. demonstrated, in randomized controlled trials, that testosterone replacement therapy (TRT) could reduce BF % in both infancy and childhood [19,21]. Data on this topic are not unequivocal and, therefore, more studies are needed [19,21].

The findings of increased amounts of body fat in prepubertal boys suggest the weakness of the hypothesis that hypogonadism and low testosterone precede obesity. The multivariate analysis highlighted that abdominal obesity is primarily responsible for the decrease in insulin sensitivity, even when serum testosterone levels are in the normal range [31]. In fact, leptin, a biomarker of the total body fat amount, is greatly elevated [29]. The temporal occurrence of events is unknown, so it is not possible to assess whether hypogonadism is responsible for the increase in abdominal adiposity or whether increased truncal fat leads to reduced testosterone production.

Perhaps there is a self-perpetuating vicious circle between obesity and hypogonadism, in which increased abdominal fat reduces the insulin sensitivity of cells, in particular Leydig cells, thus causing a progressive decrease in testosterone production [29].

Moreover, there is a possible genetic influence of body fat in KS in early childhood due to the overexpression of X-linked genes, skewed X-chromosome inactivation or CAG-repeat polymorphism in the androgen receptor as well as the influence of hypogonadism after puberty [32]. Furthermore, in a mouse model of KS, it was evidenced that only the owned number of X-chromosomes and not the differential levels of gonadal hormone influence fat storage and food intake, leading to obesity [33].

In conclusion, even though not all of the KS population develops obesity, unfavourable body composition is common and represents a key risk factor for morbidity [19,21].

### 3.2. Diabetes

Many studies have demonstrated an association between KS and diabetes, in both adulthood and childhood, although the risk factors have not been fully elucidated [13,17]. This theory is supported in part by epidemiologic data on mortality and hospitalizations in patients with KS, where the mortality rate from diabetes is significantly increased [31].

Interestingly, type 2 diabetes (T2D) has some specific features in these patients. Diabetes seems to occur earlier in life compared to the healthy population, around the age of 30 years; it shows lower insulin sensitivity and higher insulin secretion [34]; blood glucose may not be well controlled despite insulin administration; it affects patients with a low BMI or within the normal range at the diagnosis, unlike the typical non-KS-subject with T2D [35]. In adult patients affected by KS, the fasting serum insulin and fasting plasma glucose were higher, whereas insulin sensitivity was significantly reduced [31]. Moreover, the increased islet β-cell secretion function was statistically significant compared with those patients with hyperglycaemia but without KS [31].

Using the hyperinsulinemic euglycemic clamp test, Lee et al. reported that impaired peripheral insulin resistance was the underlying mechanism of impaired glucose tolerance in adult Korean patients with KS [34]. Accordingly, Yesilova et al. reported that one third of the adult KS patients have an increased incidence of insulin resistance (IR) and hyperinsulinemia [36].

We do not know when the glucose metabolism impairment exactly starts; however, it seems reasonable to suppose that early adolescence is the most critical period.

Davis et al. studied a group of adolescents with KS without a diagnosis of T2D and healthy controls between 10 and 17 years. They reported a higher HbA1c in KS patients than in healthy adolescents, which is indicative of chronically higher blood glucose [16]. More studies are needed in this field to support this hypothesis.

As previously reported, hypogonadism is an independent risk factor for the development of diabetes in men with normal chromosomes [37,38,39]; however, the prevalence of T2D is higher in Klinefelter patients than in idiopathic hypogonadotropic hypogonadism, even though both are androgen deficient and receive testosterone replacement therapy, thus suggesting that this relationship is independent of serum testosterone level [13], and this could be an association rather than the cause.

However, the chromosomal abnormality could act through other mechanisms in triggering IR, and these could be studied in KS boys at a prepubertal age. Indeed, they have similar circulating plasma levels of testosterone and gonadotropin at a prepubertal age to healthy age-matched controls of the same age despite presenting the genetic alteration typical of this disease since birth [20]. Accordingly, Bardsley et al. reported that, among 89 boys younger than 12 years old, 24% of them were insulin-resistant (according to HOMA-I) [17].

Furthermore, autoimmunity is also frequent in KS. In support of this speculative hypothesis, a recent study showed that immunoreactivity directed against diabetes-specific autoantigens of type 1 diabetes was significantly higher in KS (8.2%) than in paired controls. However, the endocrine autoantibody profile of testosterone-treated and untreated KS patients was not different [40]. Moreover, the worse the karyotype, the higher the prevalence of diabetes, probably because of the presence of an extra-X-chromosome [13].

Other mechanisms, such as changes in body composition, inflammation status, high triglyceride levels, fatty liver and acute pancreatitis, might also play an important role in the development of diabetes mellitus in KS patients [13].

However, the specific pathogenesis has yet to be elucidated, and further research is needed to clarify the complex interaction between genotype and metabolic phenotype in KS patients [41].

### 3.3. Metabolic Syndrome

Recent studies have evaluated MetS’ prevalence and occurrence since paediatric age in KS children.

Bardsley et al. investigated the risk factors for MetS in 89 prepubertal KS boys age 4–12 years [17]. Since different definitions of metabolic syndrome in childhood subsist, the authors applied the metabolic syndrome criteria set by the Third National Health and Nutrition Examination Survey [42]. In particular, MetS was defined as ≥3 of the following: fasting TG ≥ 100 mg/dL; HDL < 50 mg/dL; WC > 75° percentile for age; systolic/diastolic BP > 90° percentile and FBG ≥ 100 mg/dL. Overall, 8% of the children met the three criteria required for diagnosis, while 36% met two features of MetS. Thus, it appears that, in this prepubertal cohort of boys with KS, the risk for metabolic syndrome is already increased [17].

Similarly, Davis et al. studied the relationship between gonadal and metabolic functions in a cohort of 93 prepubertal children (4–12 years) [18]. Despite a normal BMI percentile in 95% of the subjects, at least one feature of MetS was present in 79%, while full MetS criteria were met in 11% according to previous criteria [42]. The risk of MetS was independent of age and body mass index. Moreover, the authors evaluated gonadal function via INHB, a biomarker of Sertoli cells dysfunction. They found that an INHB cut-off of ≤50 ng/dL yields a sensitivity of 83.3% and specificity of 79.2% for meeting the full criteria for MetS in boys <9.5 years of age. Thus, pre-pubertal children with the highest risk of meeting MetS criteria were those with lower values of INHB (≤50 ng/dL) [18]. As regards the lipidemic profile of these patients, adult studies describe an increase in plasma lipids, including LDL cholesterol, while HDL cholesterol is generally decreased [15].

Subsequently, the same authors hypothesized that exogenous androgen treatment would have a favourable effect on the cardio-metabolic health of KS children age 4 to 12 years from the same cohort. Thus, a randomized controlled trial with oxandrolone (Ox) versus placebo treatments was conducted. Even though a beneficial effect of Ox was appreciated for some features of MetS, after two years, the number of children who met the criteria for MetS did not differ between the groups [19].

In line with previous studies, the increased prevalence of MetS (≥3 criteria) among KS adolescents age 10–18 years was confirmed in 30% of the subjects [16].

Clarifying the risk for MetS among KS children and adolescents is crucial as features of MetS that present during childhood may track into adulthood [17]. In fact, Bojesen et al. reported that 44% of KS men (vs. 10% of controls) had MetS, associated with significantly lower concentrations of androgens, HDL cholesterol and insulin sensitivity and significantly higher levels of total fat mass, LDL cholesterol and triglycerides [11,31].

Overall, both the high prevalence of MetS and the risk of MetS independent from BMI in KS children need to be confirmed by broad cohort studies. Moreover, the impact of TRT on metabolic health during paediatric age calls for further study.

### 3.4. Cardiovascular Risk

Past epidemiological studies have found that men with Klinefelter syndrome have higher morbidity and mortality due to several conditions, including cardiovascular disease (CVD) [10,11,43]. Thus, individuals with certain genetic conditions, including KS, appear to be more susceptible to an unfavourable cardiometabolic profile. Since cardiometabolic alterations seem difficult to reverse in adulthood, it is necessary to evaluate cardio-metabolic health in KS at a younger age [16,18].

Recently, a cohort study evaluated the cardio-metabolic risk in KS adolescents age 10 to 18 years compared to age- and BMI-matched healthy controls [16]. To identify children at risk of CVD, they applied metabolic syndrome criteria [42]. The KS group showed a 96% prevalence of at least one cardio-metabolic (CM) risk feature. Overall, the KS adolescents showed a 2.5 times greater risk of having “three or more CM risk features” compared to their peers with similar BMIs. In addition, within the KS group, BMI z score positively correlated with systolic blood pressure percentile (r = 0.50; *p*< 0.001) and triglycerides (r = 0.32, *p* = 0.03). Although underpowered, the authors performed a subgroup analysis regarding the presence or absence of TRT in KS adolescents. Those receiving TRT showed significantly lower systolic blood pressure percentile (*p* = 0.033) and approximately half of the prevalence in “three or more CM risk features” compared to those who were not on TRT (18% vs. 38%), even though it was not significant (*p* = 0.13). More interestingly, this study highlights how adolescents with normal-range BMI z scores (controls 0.36 ± 1.2 vs. KS 0.31 ± 1.3) might already have an increased CM risk compared to controls [16].

Overall, an increase in cardiometabolic risk markers was also confirmed in pre-pubertal children with KS [17,18,19]. Moreover, an association between impaired gonadal functioning, evaluated via Sertoli cell markers, and cardiometabolic risk was found in KS boys age 4 to 12 years [18].

Besides cardio-metabolic outcomes, there is evidence that KS is associated with an increased risk of congenital heart malformations [11], although their frequency is still not particularly high [44]. Different congenital cardiovascular anomalies have been observed among KS adults, including transposition of the great arteries, patent ductus arteriosus, partial atrioventricular canal defect and cleft of the anterior leaflet of the mitral valve, tetralogy of Fallot and hypertrophic cardiomyopathy with sick sinus syndrome and coronary arteriovenous fistula [44,45].

Considering the role of inflammation in increasing CV risk, future studies on paediatric populations should also evaluate inflammatory biomarkers as CRP concentrations. These were found to be significantly higher in KS adults compared to controls, yet testosterone therapy has a potential role in reducing them [31]. Moreover, the role of circulating endothelial progenitor cells should be elucidated since alterations were observed in KS patients [3,16,45,46].

Overall, such CV abnormalities, together with an increased prevalence of metabolic disorders, may contribute to the increased CV morbidity and mortality observed in these patients. These data emphasize the importance of a dedicated analysis of CV in the management of patients with KS.

### 3.5. Bone Metabolism

Testosterone plays an important role, both in achieving peak bone mass by increasing serum levels during the critical period of puberty and in maintaining proper bone density, through the persistence of normal serum levels during adulthood [47]. Aksglaede et al., in a retrospective cross-sectional study of children affected by KS with a median age of 11 years old, reported normal bone mineralization and no statistical differences between testosterone-treated and untreated boys. The lumbar bone mineral content (BMC) and whole-body BMC, measured by DEXA, result within the normal range. Moreover, the serum level of testosterone and SHBG, which reflects metabolic activity, result in the lower-normal range [20]. Thus, impairment of testosterone secretion is not a prominent feature of KS boys during puberty, in contrast to adulthood, and only a few studies on KS boys describe a decrease in testosterone secretion before puberty [24,25]. Therefore, the decrease in bone mineral density should occur after puberty. In fact, adult males with KS develop delayed bone mineralization and increased bone turnover [47], increasing the risk for premature osteopenia and osteoporosis [11]. Studies in adult men with KS also described an increased risk of impaired bone mineral density and a higher morbidity and mortality from femoral and spine fractures [10,48]. Furthermore, even if the relation between bone mineral density (BMD) and serum testosterone is still not clear, low BMD has been found in untreated or delayed-treated KS patients [49]. In KS adults, the BMD was universally lowered in hip, spine and forearm regions [2], associated with decreased muscle strength and physical fitness [50,51].

Androgens seem to be important in the regulation of bone mineral content, although the exact mechanism is not yet known and, moreover, they do not seem to be solely responsible. Hypogonadism, in fact, is more relative than absolute [31]: a reduced bone mass has been demonstrated even in adult KS patients with normal testosterone levels [52], suggesting that bone loss in KS might be, at least in part, independent of the presence of hypogonadism or that the serum testosterone level might only partially define hypogonadism. Indeed, some authors have suggested that hypogonadism might be better defined using a bivariate LH and testosterone chart [53]. Unfortunately, there are no definitive data in the literature clearly showing the prevalence of osteoporosis in KS subjects with low or normal testosterone levels, or with or without other signs of hypogonadism. Other possible mechanisms could be involved in the development of bone loss in KS.

Interestingly, in a mouse model, it has been suggested that the extra X chromosome may itself cause abnormal bone structure in mice 12 to 34 months of age [54]. Another possible modulator of bone metabolism in KS could be a low oestrogen serum level and 25-hydroxyvitamin D level in the lower-normal range [55]; also, insulin-like factor 3 (INSL3), a bone-anabolic protein produced by the Leydig cells, is reduced in KS. It is correlated to osteocalcin, a marker of bone anabolic formation, and plays a role in bone metabolism by acting on osteoblasts [56]. In line with these findings, more studies are needed to shed light on the effective role of these factors in regulating bone mineral content.

## 4. Nutritional Aspects

As previously shown, metabolic and cardiovascular risks in KS patients are an important issue that may occur even during paediatric age. Obesity, diabetes, dyslipidaemia and metabolic syndrome are usually referred to as non-communicable diseases (NCDs) since they are non-infectious diseases that progress slowly but become chronic [57,58,59]. These pathological conditions seem difficult to reverse once established; thus, it is important to prevent their flourishing by establishing healthy behaviours as early as possible. In this regard, KS patients are more likely to develop NCDs compared to the healthy population [16,17,18,19,45], and prevention is crucial throughout life.

According to the Guidelines on Klinefelter Syndrome from the European Academy of Andrology (EAA), it is highly recommended to provide the patients correct information on lifestyle intervention to minimize cardiovascular risk factors, to monitor cardiovascular risk factors regularly and to treat obesity, diabetes and dyslipidaemia [60]. Counselling to provide anticipatory guidance for establishing a healthy diet and regular exercise is imperative from an early age in KS subjects [1]. The adoption of a well-rounded diet for all boys with KS should be underlined [16] to ensure balanced nutritional requirements and an adequate intake of macro- and micronutrients. Moreover, daily moderate-to-vigorous physical activity should be emphasized in accordance with recommendations for all children by the World Health Organization [61,62].

Once obesity or MetS are present, a referral to weight management programs is recommended [1]. In this case, it is necessary to adopt a multidisciplinary intervention that follows clinical guidelines for childhood obesity and metabolic syndrome treatments [63,64,65].

Regarding bone mineralization, the EAA suggests, after determination of vitamin D blood levels, an adequate vitamin D and calcium supplementation in pre-pubertal children with KS [60]. However, diet plays a role in calcium and, to a lesser extent, vitamin D intake [66,67]. Thus, nutritional advice has to take into account this aspect [1], whereas lifestyle intervention should address the importance of physical activity and sun exposure while discouraging smoking.

Considering testosterone deficiency in KS children and adolescents, deepening the knowledge of the link between diet and testosterone might be interesting. Recently, few studies have evaluated the association between dietary patterns and testosterone levels among the adult population [68,69]. Hu et al. suggest that individuals who prefer Western-style food (bread and pastries, dairy products and desserts), eat out and eat fewer homemade foods, noodles and dark green vegetables are more likely to have an unhealthy body composition (e.g., increased visceral fat) and low serum total testosterone levels, and are likely to develop hypogonadism. In fact, individuals with the highest Western scores (Q4) had a 5.72-fold (OR: 5.72; 95% CI: 1.11~29.51, *p* < 0.05) higher risk of developing hypogonadism compared to those with the lowest (Q1) [68].

The relationship between the male sex hormone, testosterone, and obesity is complex, and dietary-related factors may serve as important intermediates [68]. However, there is still substantial uncertainty regarding the role of dietary components in modifying circulating levels of testosterone [69]. Overall, randomized controlled trials are needed to confirm that an improvement in dietary pattern can improve testosterone levels and reduce hypogonadism, particularly in paediatric-age patients.

## 5. Hormonal Therapy

Even though testosterone treatment is recommended by most endocrinologists starting from the peripubertal period, no randomized placebo-controlled trials have ever been conducted [3,4,7].

Most of the studies on the outcome of TRT are observational studies that compared Klinefelter patients to healthy controls [49]. Almost all these studies focus on adults or adolescent patients. The positive effects of hormonal treatment are described by non-randomized trials in adults, which demonstrated that testosterone improves strength and endurance, fatigue, concentration and learning abilities, irritability and relations with others [2]. Moreover, an improvement in sexual function and an increase in libido have been described. Testosterone therapy appears to play a positive role in abdominal adiposity and weight loss, insulin sensitivity, insulin secretion and glucose uptake by muscle cells [3,12]. Finally, TRT also has a positive effect on bone mass density, in particular at the lumbar level [49].

There are only a few studies regarding testosterone treatment in children. Samango-Sprouse et al. evaluated the long-term neurological outcome of three months of treatment with 25 mg of testosterone in infants between 4 and 15 months of age. They found positive effects on cognitive function, in particular motor function at 6 years of age [22]. Another study evaluated the effect of administration of low-dose oral androgen in boys between 4 and 12 years old. In the end, improvements in visual–motor performance, social function, anxiety and depression were observed [23].

As partially reported above, only two studies have investigated the effect of TRT on BMI in children. One randomized controlled trial by Davis et al. showed a reduction of 15% in adiposity in 10 infants with KS treated with 25 mg of testosterone monthly for 3 months between 2 and 5 months old [21]. A second double-blind placebo versus control trial by Davis et al. showed a positive effect on metabolism and cardiovascular risk in 93 boys treated daily with low doses of oxandrolone for 2 years between 4 and 12 years old [19]. In particular, the patients treated with TRT showed a significantly lower %BF, triglycerides, blood glucose and blood pressure, both regarding prepubertal and pubertal patients. The therapy was well tolerated, although it results in advanced bone age. These studies are extremely interesting, but more evidence is required in order to suggest TRT in childhood for improving the metabolic risk in these patients. Currently, TRT is not suggested until puberty, except in the case of micropenis [70,71].

**Figure 1:**
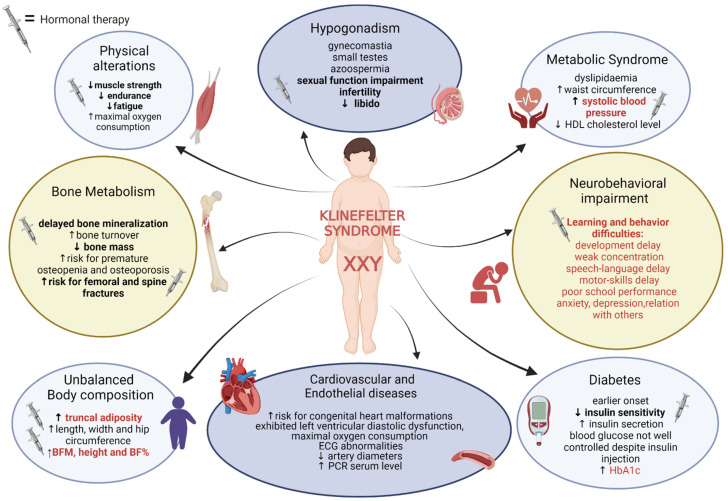
Characteristics of Klinefelter syndrome and positive effects of hormonal therapy. Bold: for features that could be corrected by TRT. Red: features studied also in children/adolescent population. Abbreviations: high density lipoprotein (HDL); electrocardiogram (ECG); C-reactive protein (PCR); body fat mass (BFM); body fat (BF). ↑ increased; ↓decreased.

Figure 1 summarizes the alterations due to KS and underlines the aspects that could be corrected by hormonal replacement therapy. Underlined in red are the paediatric features.

## 6. Conclusions

**Figure 2:**
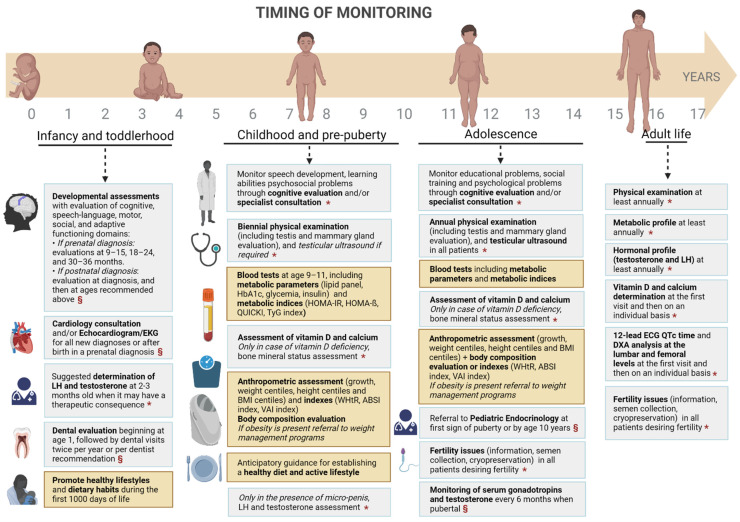
Standard (grey colour) and proposed (yellow colour) follow-up schedule of Klinefelter patients from birth to adulthood. § From reference [1]. * From reference [60]. Abbreviations: luteinizing hormone (LH); follicle-stimulating hormone (FSH); glycated haemoglobin (HbA1c); homeostatic model assessment for insulin resistance (HOMA-IR); homeostatic model assessment beta (HOMA-β); triglyceride index (TyG index); body mass index (BMI); waist-to-height ratio (WHtR); a body shape index (ABSI); visceral adiposity index (VAI index); electrocardiogram (ECG); dual-energy X-ray absorptiometry (DXA).

From the review of the literature, it appears clear that Klinefelter patients are at an increased risk of mortality, especially for metabolic abnormalities. As described above, these alterations are present since infancy, and they might be only partially explained by hypogonadism. Probably, the syndrome itself is a causative factor for genetic elements that are not yet fully understood. Considering the relevance of metabolic alterations that emerged in this review, we suggest to consider performing regular anthropometric assessments and health education to promote a healthy lifestyle since the paediatric age in addition to the evaluations recommended by the current guidelines on the management of Klinefelter syndrome [1,60] (Figure 2).

Finally, further studies are needed to better understand the etiopathogenesis of the metabolic alterations in this genetic disease. Moreover, more studies should be focused on the paediatric population in order to arrange early interventions that could improve the natural course of the disease. Table 2 summarizes the gaps and needs for future research.

## Figures and Tables

**Table 1:** Summary of studies evaluating metabolic aspects in paediatric patients affected by Klinefelter syndrome.

Authors	Type of Study	Population	Intervention or Objective	Results
Diabetes				
Davis et al. 2020 [16]	Cross-sectional study	50 KS adolescents age 10–18 years	evaluate cardio-metabolic risk in KS adolescents compared to 50 age- and BMI-matched healthy controls. Subgroup analysis performed in regard of TRT therapy	HbA1c was significantly higher in KS patients, indicative of chronically higher blood glucose
Bardsley et al. 2011 [17]	Observational prospective study	89 prepubertal KS boys age 4–12 years	compare auxologic measures and truncal obesity in prepubertal boys with KS versus age-matched controls (*n* = 34)	Truncal obesity, insulin resistance and metabolic syndrome were present in KS boys as young as 4–12 years, and these occurred in association with reduced running-type activity 24% of the boys with KS had had IR
Metabolic syndrome				
Bardsley et al. 2011 [17]	Observational prospective study	89 prepubertal KS boys age 4–12 years	compare auxologic measures and truncal obesity in prepubertal boys with KS versus age-matched controls (*n* = 34)	BMI measurements were similar, but waist circumference was >90 percentile in 30% of boys with KS versus 21% of controls. 8% of children met the three criteria required for MetS diagnosis 36% met two features of MetS 37% of the boys with KS had elevated LDL cholesterol
Davis et al. 2016 [18]	Observational study	93 pre-pubertal boys with KS age 4–12 years	assess the relationship of gonadal and cardiometabolic function in children with KS	80% of children had ≥1 feature of metabolic syndrome * (MetS) and 11% had ≥3 features of MetS * risk of MetS was independent of age and BMI. 18% had an INHB below the normal range, and a low INHB was associated with higher FBG, triglycerides, LDL and lower HDL (*p* < 0.05). INHB <50 ng/dL yielded a sensitivity of 83.3% and a specificity of 79.2% for having ≥3 features of MetS in boys <9.5 years of age
Davis et al. 2017 [19]	Double-blind RCT	79 pre-pubertal boys with KS age 4–12 years	children were randomized to receive oral oxandrolone (Ox) 0.06 mg/kg/d (*n* = 38) or placebo (*n* = 41) for 2 years.	Ox resulted in lower TG (*p* = 0.043) but also HDL cholesterol (*p* = 0.001) The number of children who met criteria for MetS * did not differ between groups at 2 years
Cardiovascular risk				
Davis et al. 2020 [16]	Cross-sectional study	50 KS adolescents age 10–18 years	evaluate cardio-metabolic risk in KS adolescents compared to age- and BMI-matched healthy controls. Subgroup analysis performed in regard of TRT therapy	KS group showed a 96% prevalence of at least one cardio-metabolic (CM) risk feature KS adolescents showed 2.5 times greater risk of having “three or more CM risk features” compared to their peers with similar BMI. KS adolescents with normal range BMI z score (controls 0.36 ± 1.2 vs. KS 0.31 ± 1.3) might already have an increased CM risk compared to controls In KS group, BMI z score positively correlated with systolic BP percentile (r = 0.50; *p* < 0.001) and triglycerides (r = 0.32, *p* = 0.03). TRT subgroup showed lower systolic BP percentile (*p* = 0.033) and approximately half of the prevalence in “three or more CM risk features” vs. not treated with TRT (18% vs. 38%), although with no significance (*p* = 0.13).
Bone metabolism				
Aksglaede et al. 2007 [20]	Retrospective cross-sectional study	24 children with a median age of 11.0 years (range 4.3–18.6)	18 untreated; 6 received oral testosterone undecanoate (40 mg twice daily increasing to 80 mg twice daily) for a median period of 1.3 years	Weight, BMI and LBM did not differ from age-matched controls, whereas height, BFM were significantly increased. No difference between treated and untreated patients with KS. Lumbar BMD and whole-body BMC were normal, indicating normal bone mineralization in both treated and untreated boys and adolescents.
Testosterone replacement therapy and adiposity				
Davis et al. 2017 [19]	Double-blind, placebo-controlled RCT	93 boys age 4–12 years	Administration of oral oxandrolone (0.06 mg/kg/day) or placebo for 2 years	%BF SDS at 2 years was significantly lower in the treatment (0.29 ± 0.76 SDS) compared with placebo group (0.81 ± 0.72 SDS) after adjusting for age and baseline %BF SDS. TRT improved cardiometabolic markers; in fact, %BF at 2 years was significantly lower in treatment group; however, it caused lower HDL cholesterol and advanced bone age
Davis et al. 2019 [21]	Prospective randomized trial	20 infants, 6–15 weeks of age	Administration of 25 mg testosterone cypionate intramuscular monthly for three doses vs. no treatment	Testosterone treatment resulted in positive changes in body composition
Testosterone replacement therapy and cognitive function				
Samango-Sprouse et al. 2013 [22]	Placebo-controlled RCT	101 children 36–72 months of age	Administration of injections (25 mg each) of testosterone enanthate, or placebo. 1 injection/month for 3 months 34 treated, 67 untreated	There were significant differences in multiple cognitive domains in the group that received androgen treatment, including multiple measures of language, intellectual and neuromotor skills.
Ross et al. 2017 [23]	Placebo-controlled RCT	84 children age 4–12 years	Administration of oxandrolone (0.06 mg/kg daily) or placebo for 24 months 43 treated, 41 untreated	Benefited visual–motor function and positive effects on anxiety, depression, social problems
Hypogonadism and hormonal aspects				
Lahalou et al. 2004 [24]	Observative prospective study	18 KS infants from birth to 3 years	Blood samples were collected from birth to 3 years of age and compared with those in 6 adolescents (14–18 years) with XXY karyotype and reference values established in 215 control infants	In XXY infants, FSH, LH, INHB and AMH did not differ from controls Testosterone levels during the first trimester exhibited a physiological increase but were lower than in controls (*p* < 0.0001). Significant correlations were found between testosterone and LH (*p* < 0.001), between INHB and FSH (*p* = 0.0011) and between AMH and INHB (*p* = 0.025). In XXY adolescents, AMH and INHB were undetectable. Testosterone secretion is impaired in infants with Klinefelter syndrome. By contrast, INHB and AMH secretions were not altered
Ross et al. 2005 [25]	Observative prospective study	22 infants and young boys with KS, age 1–23 months	Auxologic measurements, biologic indices of testicular function by blood samples and clinical assessment of muscle tone in KS infants were measured.	Mean length, weight and head circumference in SDS were generally within the normal range at –0.3 ± 1.0, −0.1 ± 1,4 and 0.0 ± 1.5, respectively. Mean penile length and testicular volume SDS were –0.9 ± 0.8 and –1.1 ± 0.8, indicating significantly reduced penile and testicular size. Mean testosterone levels for the boys < 6 and > 6–23 months were 128 ± 131 (4.4 + 4.5 nmol/L) and 9.5 ± 7.2 ng/dL (0.3 ± 0.2 nmol/L), respectively. Hypotonia was present in 12/17 boys

Klinefelter syndrome (KS); randomized controlled trial (RCT); body mass index (BMI); body fat percentage (%BF); standard deviation score (SDS); lean body mass (LBM); body fat mass (BFM); testosterone replacement therapy (TRT); insulin resistance (IR); metabolic syndrome (MetS); inhibin B (INHB); waist circumference (WC); triglycerides (TG); glycated haemoglobin (HbA1c); low density lipoprotein (LDL); high density lipoprotein (HDL); fasting blood glucose (FBG); blood pressure (BP); oral oxandrolone (Ox), bone mineral content (BMC); bone mineral density (BMD); follicle-stimulating hormone (FSH); luteinizing hormone (LH); anti-Mullerian hormone (AMH); inhibin B (INHB). * MetS defined as ≥ 3 of the following: WC > 75% for age, fasting FG > 100 mg/dL, HDL < 50 mg/dL, FBG > 110 mg/dL and systolic or diastolic BP > 90% for age and height.

**Table 2:** Future research gaps and needs in KS.

Future Research Gaps and Needs in Klinefelter Syndrome		
Metabolic and Hormonal Aspects	Nutritional Aspects	TRT
Clarifying the complex interaction between genotype and metabolic phenotype in KS paediatric patients Timing of beginning of glucose metabolic impairment and its specific pathogenesis Clarifying the relationship between testosterone production, truncal adiposity and hypogonadism Broad cohort studies should determine prevalence of MetS among KS children and whether it is independent from BMI Identification of early cardiovascular biomarkers to monitor and evaluate CV health in KS children Effects of 25-hydroxyvitamin D, INSL3 and osteocalcin on regulating bone mineral content	Prospective evaluation of the potential impact of healthy lifestyles on growth and metabolic health in KS children Development of targeted nutritional strategies Deepen the knowledge of the link between diet and testosterone	Timing of beginning of TRT Effect of TRT on metabolic alteration and outcome in children

Klinefelter syndrome (KS); body mass index (BMI); metabolic syndrome (MetS); testosterone replacement therapy (TRT); cardiovascular (CV).

## Data Availability

Not applicable.
